# Tomographic aspect of a giant stone in a bricker urinary diversion

**DOI:** 10.1590/S1677-5538.IBJU.2024.9919

**Published:** 2024-08-10

**Authors:** Luciano A. Favorito, Arthur Valdier, André L. Diniz, Ana Raquel M. Morais, José A. de Resende

**Affiliations:** 1 Hospital Federal da Lagoa Rio de Janeiro RJ Brasil Serviço de Urologia, Hospital Federal da Lagoa, Rio de Janeiro, RJ, Brasil; 2 Universidade do Estado do Rio de Janeiro Unidade de Pesquisa Urogenital Rio de Janeiro RJ Brasil Unidade de Pesquisa Urogenital, Universidade do Estado do Rio de Janeiro - UERJ, Rio de Janeiro, RJ, Brasil

## COMMENT

**Figure 1:**
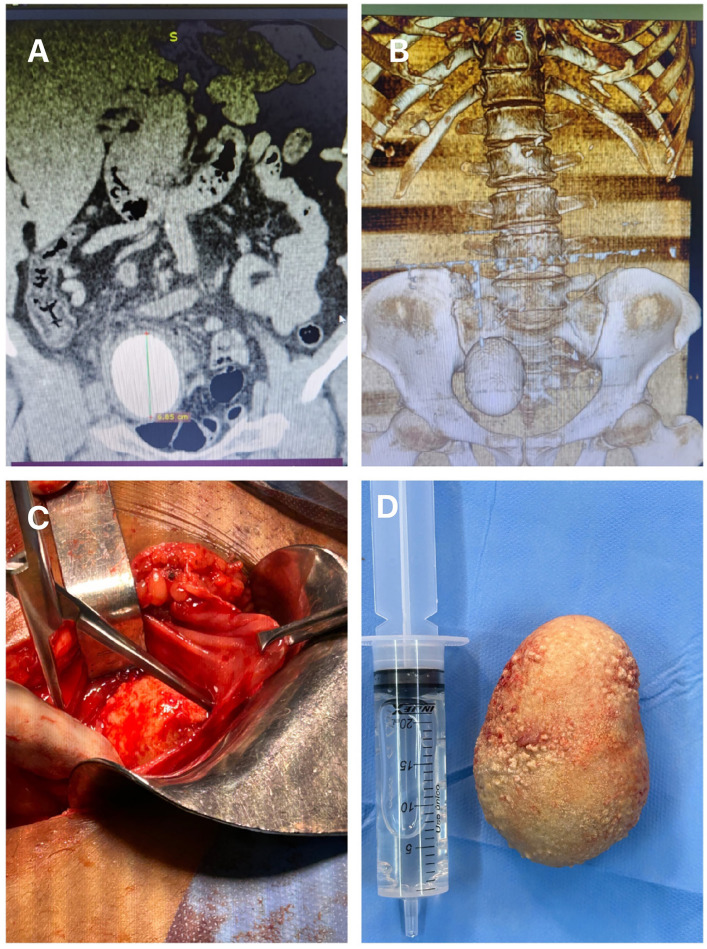
A) The figure shows the CT with the measurement of the stone inside the urinary diversion; B) In this figure we can observe a CT reconstruction showing the aspects of the Bricker stone; C) The figure shows the access to the Bricker to remove the stone and D) The figure shows the stone with 6.6cm removed after the open surgery.

Robotic cystectomy has become increasingly popular for the treatment of muscle invasive bladder cancer, but open cystectomy is the gold standard treatment for this disease (1, 2). The ileum is used as a conduit to drain urine to the abdominal wall as a urinary stoma after radical cystectomy usually (Bricker urinary diversion) (1, 2). There are some complications after the radical cystectomy with Bricker reconstruction and the urolithiasis is one of the most common (3–5). Many factors contribute to stone formation, being urinary stasis, mucus production and bacteriuria the most important (3, 4). One of the techniques to treat urolithiasis in Bricker diversion is the open surgical removal, mainly in large stones (3–5). In this paper we present a 65-year-old patient with a large stone inside of Bricker, 5 years after radical cystectomy for the treatment of muscle invasive bladder cancer. The patient had pain and urinary infection with fever. The CT shows a stone inside the Bricker measuring 6.5cm (Figure-1). The patient was submitted to open laparotomy to remove the stone inside the Bricker. The stone weighted 670g (Figure-1). The patient had excellent evolution after the procedure.
